# Frequency and features of nocturnal enuresis in Pakistani children aged 5 to 16 years based on ICCS criteria: a multi-center cross-sectional study from Karachi, Pakistan

**DOI:** 10.1186/s12875-018-0876-5

**Published:** 2018-12-14

**Authors:** Sameena Shah, Rabab Zehra Jafri, Khalil Mobin, Rabeea Mirza, Kashmira Nanji, Fatima Jahangir, Sufian Jawed Patel, Muzamil Shabana Ejaz, Iman Qaiser, Hira Iftikhar, Komal Aziz, Wajiha Khan, Humza S. Maqbool, Hassaan Ahmed

**Affiliations:** 1 Department of Family Medicine, Aga Khan University, Stadium Road, Karachi, Pakistan; 2 Massachusetts General Hospital for Children, Harvard Medical School, Boston, MA USA; 3 Community Health Sciences, Karachi Medical and Dental College, Karachi Metropolitan Corporation, Karachi, Pakistan; 4 Cleveland Clinic, Cleveland, Ohio USA; 5 Department of Family Medicine, Aga Khan University, Karachi, Pakistan; 6 Department of Family Medicine, Ziauddin University Hospital, Karachi, Pakistan; 7 Department of Medical Oncology, Aga Khan University, Karachi, Pakistan; 8 Dow Medical College, DOW University of Health Sciences, Baba-e-Urdu Road, Karachi, Pakistan; 9 Mercy Health, Grand Rapids, Michigan, USA; 10 Beaumont Health Royal Oak, Michigan, USA; 11 Saint Louis University, St Louis, MO USA; 12 Department of Pediatrics, Aga Khan University, Karachi., Pakistan; 13 UC, San Francisco - Fresno medical education program, California, USA; 14 South Tyneside District Hospital, South Shields, UK

**Keywords:** Nocturnal enuresis, Symptoms, Co-Morbids, LUTS, Children, Cross-sectional analysis, Pakistan, International Children’s continence society

## Abstract

**Background:**

Nocturnal enuresis (NE) is a common symptom in children worldwide. International Children’s Continence Society (ICCS) defines enuresis as either mono-symptomatic, NE with lower urinary tract symptoms and NE with co-morbid conditions. The objectives of this study were to determine the frequencies and types of NE and associated symptoms and conditions in children aged 5 to 16 years based on ICCS criteria.

**Methods:**

A multi-center cross sectional study was conducted between November 2012 and December 2013 in the primary care clinics of four hospitals in Karachi. Children aged five to fifteen years were included through consecutive sampling. Informed consent was obtained from the parents and a pre-coded semi-structured questionnaire was used to obtain the information. Data was entered on SPSS version 20.0 and multivariable logistic regression analysis was used for data analysis.

**Results:**

Out of 429 children aged between five and sixteen years, 243(56.9%) were boys and the remaining 186(43.1%) were girls. One hundred and eighty three children (43%) had nocturnal enuresis (NE). Forty four (10.3%), had mono-symptomatic NE, 57(31.1%) had associated lower urinary tract symptoms (NE-LUTS), whereas 30 (16.3%) had NE with a co-morbid condition. Fifty two (28.4%) NE’s had at least one of both LUTS and a co-morbid condition. Out of the 246(57%) non-enuretic’s, 31(12.6%) had a LUTS, 95(38.6%) had a co-morbid condition and 57(23.2%) had at least one of both LUTS and a co-morbid condition. The remaining 63 (25.6%) were symptom free. Increased voiding frequency, urgency, dysuria, suprapubic pain and daytime incontinence were the LUTS significantly associated with NE. Co-morbid conditions significantly associated with NE included constipation, congenital defects, developmental delay, and learning and sleep problems.

**Conclusion:**

Although NE can be an only symptom, it is often associated with lower urinary tract symptoms like dysuria, urgency, suprapubic pain, and daytime incontinence. Children presenting with NE often have co-morbid conditions like constipation, urinary tract infection, sleep disorders, and developmental delay. Many children presenting with these conditions as the primary complaint may also have NE. It should be addressed as unrecognized and untreated NE can cause additional morbidity and distress.

## Introduction

Enuresis or nocturnal enuresis (NE) in children aged five to sixteen years is seen worldwide in all cultures and across all socioeconomic strata [1]. A close connection exists between nocturia in adulthood (parents) and enuresis in childhood [2, 3]. The literature is rife with a multitude of definitions and descriptions of symptoms of involuntary passage of urine in children and adolescents [1]. The reported prevalence rates of this symptom vary according to the definition selected and the age group of children surveyed [1]. This has made it difficult to compare the various aspects of NE across age groups, cultures and regions [4]. However, it is possible that the true incidence of NE is unknown due to under reporting [5].

According to the current International Children’s Continence Society (ICCS) definition, “any kind of wetting episode that occurs in discrete amounts during sleep is called enuresis or NE, regardless of the presence of concomitant daytime symptoms or any other symptoms that may be present” [1]. This definition is applicable from the age of five years as recommended by the ICCS, the World Health Organization (WHO) and American Psychiatric Association’s ‘Diagnostic and Statistical Manual of Mental Disorders, 5^th^ Edition’ (DSM V) [1, 6, 7]. According to the ICCS, NE is a symptom and a condition synonymous with intermittent nocturnal incontinence. It means incontinence in discrete episodes while asleep. It is classified as ‘primary’ if the child has previously been dry during sleep for less than six months and ‘secondary’ if the child started bed wetting after remaining dry for at least six consecutive months [1].The ICCS does not define the duration for which a child has to be enuretic before being judged as such. The DSM-V defines it as 3 months of bedwetting accidents for a child to qualify as an enuretic and requires wetting for at least 2 nights per week for a child who has reached the chronological age of 5 years at least and/or a mental age of 5 years [7]. The World Health Organization, International Classification of Disease, Tenth Edition (WHO ICD-10), requires at least 2 episodes of involuntary urination per month for children between the ages of 5 and 7 years and at least 1 for children 7 years and above, for 3 consecutive months to qualify to have NE [6]. The prevalence rate of NE in school-based studies declines by age as the child moves into adolescence. This is attributed to the natural process of spontaneous resolution as the child matures and occurs at the rate of approximately 15–20% per year so that by the age of fifteen – eighteen years it reduces to 1–2% matching the prevalence in adults [8–10]. International Children’s Continence Society categorizes it further as ‘monosymptomatic nocturnal enuresis’ (MSNE) if there is isolated NE and non-monosymptomatic nocturnal enuresis or NE with lower urinary tract symptoms (NE-LUTS), if lower urinary tract symptoms like dysuria, suprapubic pain, and daytime incontinence are also present [1]. In addition, it also clarifies the co-morbid conditions considered relevant and important in association with NE. They include constipation, encoporesis, urinary tract infection (UTI), suprapubic pain, asymptomatic bacteriuria, vesicoureteral reflux, neuropsychiatric disorders, learning disabilities and sleep disorders [1]. A predictive association between factors like ethnicity, age, gender, birth order, education level and employment status of parents, family size, income, socio-economic status, bedtime habits, arousal dysfunction, birth order and history, NE frequency and bedwetting has also been observed [4].

Epidemiological data regarding this common condition is limited in Pakistan. The objectives of the study, therefore, were to determine the frequency, types, features and conditions associated with NE in children aged 5 and 15 years based on ICCS standards of terminology and grouping criteria [1].

## Materials and methods

A cross sectional survey was conducted between November 2012 and December 2013 in the outpatient and offsite family medicine and pediatric primary care clinics of two public and two private hospitals situated in four widely separated regions of the city of Karachi, Pakistan. Approval was taken from the ethics review committees of all four hospitals. A sample size of 425 was calculated assuming a community prevalence of 50% to ensure a maximum sample size with 95% confidence interval and bound of error of 5% to fulfill the objectives of the study. The WHO software for sample size determination was used to calculate the sample size. The inclusion criteria were all children aged at least 5 years and less than sixteen years, regardless of any past or present disease, condition or complaint and whose parents were seated in the waiting room for whatever reason and who gave informed consent for data collection. The data collectors were trained to ensure uniformity in ensuring that the criteria of the ICCS definitions and terminology were adhered to accurately. A specially designed pre-coded, semi-structured questionnaire was developed in English and Urdu based on the terminology recommended by the ICCS and was cross validated before use. It consisted of dichotomous and qualitative questions covering demographic details, the wetting episodes, and associated factors like LUTS and co-morbid conditions. All parents present in the data collection sites for any reason, during clinic hours were approached by the same data collectors consecutively till the required sample size was achieved. All the information was gathered only from parents who gave informed consent. The children did not have to be present in the clinic at the time of data collection. The ICD-10 criteria for age groups and bedwetting frequency were used for this study. Five years was selected as the minimum age of inclusion because it is as the minimum age limit for NE according to the ICCS definition of NE.

Sixteen years was selected as the upper age limit for inclusion because this is the upper limit of the pediatric age group and the prevalence of bed wetting decreases to match that of adults by sixteen years of age. The parents were asked about the presence or absence of NE in their child/children and information regarding demographics, associated features and conditions was gathered according to our objectives. They were also asked if they had ever sought help or consulted a physician for NE. Children who had ever wet their beds were categorized as ‘nocturnal enuretics’ (NE) and those who had never wet their beds as non-enuretics (Non-NE). Children with only NE and no LUTS or co-morbids as defined by ICCS were designated as children with monosymptomatic NE (MSNE). Children with NE and associated lower urinary tract symptoms were categorized as (LUTS-NE). Children who had NE and at least one co-morbid were designated NE with a co-morbid. The questionnaire took approximately 20–30 min to complete. Any child found to have unrecognized and untreated enuresis or voiding abnormalities and who was not under medical care was referred to pediatricians who had given consent for this purpose. Confidentiality was maintained through coding all the personal details with access to the principal investigator only.

### Data analysis

Data was double entered and analyzed in SPSS version 20. The outcome variable in the study was NE. Frequencies were reported for all the categorical variables such as parents’ education, marital status and employment status, gender of the child, co-morbid conditions, and associated symptoms. The frequency of children with NE was compiled to meet our first objective. The frequencies of MSNE and of children with at least one LUTS or at least one co-morbid or at least one of both were calculated. Frequencies of children with at least one LUTS and at least one co-morbid but without NE were also calculated. Demographic and social characteristics were derived. Chi-square test was used to compare symptoms and bedtime micturition behaviors of children with and without NE. Multivariable logistic regression analysis was performed to identify association of various factors with NE. Univariate analysis was also performed to identify independent association of various socio demographic variables with NE. Results have been reported in the form of unadjusted odds ratio with 95% confidence interval. A 5% level of significance was used throughout the study and all analysis was carried out using two-sided tests.

## Results

### Sociodemographic variables of the children

The data were compiled and analyzed according to ICCS definitions and related terminology wherever available. Out of the final sample of 429 children aged between 5 and 16 years, 243 (56.9%) were boys and the remaining 186 (43.1%) were girls. Children aged between 5 to 7 years constituted the largest number. Father’s and mother’s educational level, father’s occupational status and socioeconomic status measured as average monthly income were the only socio-demographic variables that had significant association in the two groups. Table 1.

**Table 1:** Sociodemographic variables of Nocturnal Enuretic’s and Non-enuretic’s (*n* = 429)

Variable	Nocturnal Enuretic’s (*n* = 183)	Non-enuretic’s (*n* = 246)	Adjusted Odds Ratio (95% CI)	*P*-Value
Gender				
Boys	104 (56.8)	139 (56.5)	1.005 (0.70–1.44)	0.96
Girls	79 (43.2)	107 (44.5)	1	
Age of child				
5 to 7 years	83 (45.4)	104 (42.6)	1.146 (0.54–2.41)	0.97
8 to 10 years	55 (30)	73 (29.9)	1.097 (0.51–2.35)	
11 to 13 years	32 (17.5)	51 (20.9)	1.209 (0.54–2.69)	
> 13 years	13 (7.1)	18 (6.6)	1	
Birth Order of Child				
1st	64 (35.0)	85 (34.8)	1.091 (0.64–1.85)	0.67
2nd	58 (31.7)	72 (29.5)	0.979 (0.57–1.67)	
3rd	28 (15.3)	46 (18.9)	1.351 (0.74–2.5)	
4th	33 (18.0)	43 (16.8)	1	
Mother’s Educational Status				
Illiterate	79 (43.2)	81 (33.3)	0.583 (0.38–.898)	0.013*
Primary	29 (15.8)	29 (11.9)	0.444 (0.25–0.78)	
Secondary	27 (14.8)	40 (16.4)	0.878 (0.494–1.56)	
Intermediate & above	48 (26.2)	96 (38.5)	1	
Father’s Educational Status				
Illiterate	44 (24.0)	56 (23.0)	0.730 (0.47–1.14)	0.009*
Primary	21 (11.5)	16 (6.6)	0.419 (0.22–0.80)	
Secondary	50 (27.3)	45 (18.4)	0.514 (0.32–0.83)	
Intermediate & above	68 (37.2)	129 (52.0)	1	
Mother’s Occupation				
House wife	170 (92.9)	215 (88.1)	1	0.59
Employed	13 (7.1)	31 (11.9)	1.296 (.72–2.33)	
Father’s Occupation				
Employed	183 (100)	239 (98.0)	1	0.39
Unemployed	___	7 (2.0)	1.481 (0.35–6.26)	
Avg. monthly house hold income				
Don’t Know	17 (9.3)	42 (17.2)	1	0.007*
Less than Rs.10000	54 (3.8)	18 (7.4)	2.780 (1.33–5.78)	
Rs.5–10,000	47 (56.5)	42 (17.2)	2.598 (1.32–5.09)	
Rs.10–20,000	79 (43.2)	80 (32.8)	1.612 (0.75–3.45)	
More than 20,000	33 (18)	64 (25.4)	1.055 (0.31–3.51)	

*P-value significant at 0.05 level

### Types and characteristics of nocturnal enuresis

The frequency of NE, NE with LUTS and NE associated with co-morbid conditions was greater in boys 104(56.2%) than girls 79(43.2%) (*P* = < 0.001). Sixteen families had more than one child who was either bedwetting currently or who had wet the bed in the past and was now cured. Out of the 183 (43%) children with NE, 165 (90.2%) had primary NE and 16 (8.7%) had secondary NE. The frequency of MSNE was 44 (24%), the frequency of NE-LUTS was 57 (31.1%) and the frequency of NE associated with co-morbid conditions was 30 (16.4%). Fifty two NE’s (28.4%) had at least one each of both LUTS and a co-morbid condition. Out of the 246 (57%) non-NE’s, 31(12.6%) had at least one LUTS, 95(38.6%) had a co-morbid condition and 57 (23.2%) had at least one each of a LUTS and a co-morbid condition. The frequencies of non-NE’s without LUTS or co-morbid conditions and those with either LUTS or a co-morbid condition or at least one of each are given in Table 2**.** Parents of 88 (48%) children with NE had sought help for the problem whether from a doctor, alternative health practitioner, family member, elder or another source. Out of these only 52(28.4%) had consulted a doctor for the problem.

**Table 2:** Categories of Nocturnal Enuresis (*n* = 429)

Variable	Nocturnal Enuretics (*n* = 183)	Nonuretics (*n* = 246)
Mono only (*n* = 44)	44	–
LUTS only (*n* = 88)	57 (31.1)	31 (12.6)
Comorbid factors only (*n* = 125)	30 (16.3)	95 (38.6)
Atleast one of each (LUTS/Comorbid factors)	52 (28.4)	57 (23.2)
None	____	63

### Child and family characteristics and behaviors of the children

Chi square analysis of related variables in the sampled children revealed some significant associations with NE. These variables included consanguineous marriage, family history of NE (whether in parents, siblings or first cousins) and mistreatment/bullying of the child. Table 3**.**

**Table 3:** Comparison of lower Urinary Tract Symptoms associated with Bed wetting in Nocturnal Enuretics and Nonuretics (*n* = 429)

Variables	Nocturnal Enuretics (*n* = 183)	Nonuretics (*n* = 246)	*P*-value
*Voiding frequency during the day 8 times or more*	33 (18.0)	18 (7.4)	< 0.001*
*The child runs to the bathroom urgently*	39 (21.3)	10 (4.1)	< 0.001*
*Burning or pain on passing urine*	28 (15.3)	06 (2.5)	< 0.001*
*Presence of lower abdominal/suprapubic pain*	35 (19.1)	16 (6.6)	< 0.001*
*The child leaks urine continuously (continuous incontinence)*	19 (10.4)	–	–
*Presence of grunting or straining*	09 (4.9)	05 (2.0)	0.075
*Abnormal posturing to prevent urgency/leakage (holding maneuvers)*	32 (17.5)	27 (11.1)	0.045
*Does the child have day time incontinence (daytime incontinence)*	30 (16.4)	03 (1.2)	0.000*

*P-value significant at 0.05 level

### Comparison of lower urinary tract symptoms in nocturnal enuretics and non-enuretics

Several LUTS were found to be significantly associated with NE. They included increased voiding frequency (> than 8 times a day), daytime incontinence, urgency, dysuria, suprapubic pain, continuous incontinence and daytime incontinence. The presence of grunting or straining and abnormal posturing did not have significant association with bedwetting. Table 4.

**Table 4:** Family Characteristics and Behaviors of Nocturnal Enuretics in Comparison with Non- Nocturnal Enuretics

Variable	Nocturnal Enuretics (*n* = 183)	Nonuretics (*n* = 246)	*P*-value
*Place of delivery*			
Hospital	42 (23.0%)	53 (21.5%)	0.029*
Home	133 (72.7%)	180 (73.2%)	
Others	8 (4.4%)	13 (5.3%)	
*Full Term Pregnancy*	169 (92.3%)	224 (91.1%)	0.633
Type of Delivery			
Normal	159 (86.9%)	206 (83.7%)	0.366
Caesarian Section	24 (13.1%)	40 (16.3%)	
*Childbirth Complications*	20 (10.9%)	18 (7.3%)	0.193
*Family Size*			
< than 5	37 (20.2)	58 (23.6)	0.407
> than 5	146 (79.8)	188 (76.4)	
*The child sleeps separately*	40 (21.9)	72 (29.3)	0.08*
*There are family fights*	34 (18.6)	26 (10.6)	0.018*
*Child is bullied/mistreated*	43 (23.5)	27 (11.0)	0.001*
*The child empties her/his bladder before going to bed*	149 (81.4)	199 (80.9)	0.89
*The child drinks or eats within 2 h before going to bed*	148 (80.9)	191 (77.6)	0.44
*The toilet is close and easy to reach*	131 (71.6)	143 (58.1)	0.013*
*The child goes to bed at the same time every night*	103 (56.3)	137 (55.7)	0.903
*The child drinks any kind of Cola drink*	89 (48.6)	138 (56.1)	0.126
*Did/does a family member also wet/s their bed*	83 (45.4)	68 (27.6)	< 0.001*
*Consanguineous marriage of parents*	117 (63.9)	108 (43.9)	< 0.001*
*Relationship of parents before marriage (n = 117)*			
1st cousin	74 (63.2)	71 (65.8)	0.044*
2nd cousin	34 (29.0)	27 (25.0)	
Other	9 (7.6)	10 (9.2)	
*The mother has a medical condition*	17 (9.3)	21 (8.5)	0.786
*The father has a medical condition*	17 (9.3)	28 (11.4)	0.48
*A sibling has a medical condition*	21 (12.0)	25 (11.6)	0.04*

*P-value significant at 0.05 level

### Comparison of comorbid conditions found in nocturnal enuretics with non-enuretics

The co-morbid conditions found to be significantly associated with NE included constipation, developmental delay, congenital defects, learning problems, sleep problems, diagnosed UTI and the presence of any other co-morbid condition. Table 5.

**Table 5:** Comparison of Co-morbid Conditions found in Nocturnal Enuretics in Comparison with Non- Nocturnal Enuretics (*n* = 429)

Variables	Nocturnal Enuretics (*n* = 183)	Nonuretics (*n* = 246)	*P*-value
*Presence of constipation (either hard to pass stools or < twice per week)Yes*	42 (23.0)	29 (11.9)	0.002*
*Developmental delay*	14 (7.7)	4 (1.6)	0.004*
*Congenital Defect*	7 (3.8)	04 (1.6)	0.102*
*Physical delay*	16 (8.7)	10 (4.1)	0.010
*Allergy*	20 (10.9)	26 (10.1)	0.869
*Learning problem*	14 (7.7)	14 (5.8)	0.002*
*Asthma*	11 (6.0)	11 (4.5)	0.045
*Diabetes Mellitus*	–	–	–
*Sleep problem*	42 (22.9)	22 (9)	0.001*
*Seizures*	01 (0.5)	01 (0.4)	0.930
*Kidney or bladder problems*	4 (2.2)	–	
*Urinary tract infection*	14 (7.7)	1 (0.4)	< 0.001*
*Use of medication*	20 (10.9)	10 (4.1)	0.006
*Medical problem, condition or disease*	20 (10.9)	10 (4.1)	< 0.001*

*P-value significant at 0.05 level

## Discussion

To the best of the authors’ knowledge this is the first study estimating NE frequency and characteristics in the children of parents visiting outpatient and offsite clinics of 4 hospitals, situated in widely separated areas of Karachi, Pakistan, based on ICCS criteria and terminology. Their patient population is representative of the general population of Karachi as they serve patients from all regions including migrant communities. These clinics, in effect, function as primary care clinics as a distinct pathway from primary to tertiary care does not exist. The large majority of patients are self-referred to these clinics.

The relatively high frequency of 43.1% of NE found in this study is more likely to be accurate because these parents were interviewed in health care settings and they were there because they or their child had a health-related problem. Therefore, they were more likely to be willing to share medical information and more inclined to be accurate and honest in their responses. A similar observation has been made in two earlier papers regarding NE from Pakistan [11, 12]. Another reason could be that the questionnaire was completed by trained data collectors rather than the parent and therefore more accurate and complete. In addition, the parents were specifically and categorically asked about the presence or absence of each LUTS and co-morbid condition listed by the ICCS. Another explanation for the high frequency could be that some parents may have been attending the clinics for LUTS and co-morbid conditions like constipation, developmental delay, and congenital defects in their children, all of which have been found to be associated with NE. The parents were not seeking treatment for NE at the time although 52 (28.4%) had sought treatment for it from doctors, out of a total of 88(48%) altogether, who had visited either an alternative health care practitioner or had consulted a family elder. A greater frequency of NE in children between 5 and 7 years and above the age of 7 years could be because the more stringent ICD-10 criteria for age groups were used rather than the DSM-V criteria that would have excluded many children with NE from inclusion [7]. The sample was balanced in terms of gender at 56.9% boys and 43.1% girls. The frequency of NE in boys was significantly greater than in girls which is in keeping with multiple earlier reports in international and local studies [3, 4, 11]. Children of mothers with a higher education level had a greater risk of having NE. In our sample size, being illiterate was protective with the least odds of having a child with NE, in comparison to an increased risk of having an enuretic child with a higher level of education. The same association was found for fathers. Table 1 However, a similar association with higher educational level of mothers has been noted in earlier publications from Iran and Taiwan, both of which have much higher female literacy rates of nearly 80 and 97% respectively, compared to a female literacy rate of only 44% in Pakistan [3, 13, 14]. In contrast, some studies have reported an association of lower education level of the mother with enuresis [11, 15, 16]. Possible reasons for these mixed findings could be bias and confounding factors like variable female literacy rates, socio-economic status, rural versus urban sites, school versus community study settings and sample characteristics [11, 15]. In this study, significantly more employed mothers were likely to have enuretic children, whereas unemployment of the father was found to be a positive predictor for NE. Both these finding are supported by earlier publications [15]. On the other hand, some studies have not shown any association with employment status of either parent [17]. Further research will clarify the reasons for these mixed findings.

Internationally, and in Pakistan, a wide range of age groups, (1–18 years), have been sampled and analyzed on the basis of different definitions and descriptions yielding a wide range of prevalence’s ranging from 3.8 to 23.03% and 9.1 to 22.5% respectively [4, 11, 12, 18–20]. However, they are not comparable because the sampled age groups ranged from 1 to 18 years, the definitions of NE have not been uniform (ICD-10 or DSM-IV), the settings have ranged from community, schools, rural and urban sites, and data collection has been through direct interviews or self-administered questionnaires. This has resulted in unreliable data limiting the understanding and comparison of pediatric LUT dysfunction internationally and in Pakistan [1].To ensure validity, a minimum of 5 and maximum age of 16 years was selected for this study as before 5 years, bed wetting is considered normal and after 16 years the prevalence rates match those of adults [21].^,^ In our sample, the frequency of NE in each age group decreased by approximately 10–15% so that it became 1.5% in children aged 13–15 years and 0.5% in 15 year old’s which matches the already established rate of decrease of prevalence of NE of approximately 15–20% per year to become 1–2% by the age of fifteen–eighteen years equal to that reported in adults [22, 23]. Table 1.

In Pakistan, the prevalence rates in the two community-based studies done so far on children aged 4–14 and 6–15 years respectively, are 22.5 and 25% [11, 14]. These frequencies are higher in comparison to school-based studies done on children aged 3–13, 5–15, and 6–12 years that reported prevalence’s of 9.1,13.1 and 9.7% respectively [12, 13, 15]. The lower frequencies in school based studies may be due to erroneous documentation in self-completed questionnaires and/or incomplete or false information due to the embarrassment and shame associated with this condition compared to direct interviews by trained data collectors in the community based studies, as has been noted in earlier publications [11, 12]. Another reason could be difficulty in understanding the questions by parents when responding to self-administered questionnaires resulting in inaccurate and incomplete responses which has also been suggested in a previous publication in Pakistan [11, 12]. A community and one school based study conducted in Pakistan reported MSNE prevalence of 25 and 9.7% respectively as per ICCS definition [11, 12].The MSNE frequency of 10.1% found in the present study is similar to the prevalence of 9.7% based on ICCS criteria, reported in a school based survey and the NE prevalence’s of 13.1 and 9.1% of the remaining two school based surveys, even though they were not based on ICCS criteria [12, 13, 15].None of them distinguished between MSNE and NE-LUTS or NE with co-morbid conditions, although one of them reported the percentage of NE with dysuria, UTI, constipation and diabetes mellitus [12, 13, 15]. In comparison, prevalence’s ranging from 1.37 to 18.9% depending on the selected age groups and whether the DSM-IV or ICD-10 criteria were used have been reported in a number of school based studies in Asian countries including Iran (6.8%), China (4.20%), Malaysia (8%), Thailand (4.2%), Taiwan (5.5%), Turkey (12.4%), Korea (12.8%), India (7.6%), and Saudi Arabia (15%), respectively, although the age groups selected varied widely amongst them [3, 4, 24–28].

The frequency of children with LUTS, (with at least one LUTS), regardless of the presence of NE, was 88 (20.5%) in this sample. Of these, 31 (12.6%) were not bedwetting, but the majority, 57 (31.1%), of these children with LUTS had NE. This is in comparison with a frequency of LUTS with NE of 20% as reported previously [29, 30]. It follows that a child having LUTS is more likely to have NE than a child with a co morbid condition. Table 2 Twenty five percent children had at least one LUTS and at least one co-morbid condition. Out of these 52 (28.4%) had NE and nearly the same number, 57 (23.2%) did not have NE. Table 2.

Lower urinary tract symptoms as defined by ICCS that had significant association with NE included increased voiding frequency (> than 8 times a day), daytime incontinence, urgency, dysuria, suprapubic pain, continuous incontinence and daytime incontinence. A frequency of 14.4% of dysuria reported in an earlier study done in Karachi is very similar to the frequency of 15.3% in this study [13]. Table 4 The frequency of NE-LUTS was higher than MSNE in older age groups of children, possibly because MSNE is more likely to have resolved spontaneously in older children. A similar observation has been made in an earlier work [26].

The remaining 30(16.3%), enuretic children had at least one associated co-morbid condition with NE and 95 (38.6%) children with at least one co-morbid condition did not have NE. Of these, constipation, developmental delay, congenital defects, learning problems, sleep problems, and diagnosed UTI were significantly associated with NE. Table 5 A frequency of 23% for constipation which was significant, 7.7% for UTI and 22.9% for sleep disorders was found in this study compared to 13.4 and 6.4% and 11.8% respectively reported in a previous study done in Pakistan [13]. A statistically significant increase in NE has been noted in the presence of constipation and successful treatment of constipation can result in resolution of NE, daytime incontinence and urinary tract infection as reported earlier [31, 32]. The association of LUTS and UTI with NE is also well recognized [17, 18].

Many children with conditions like UTI, constipation, developmental delay, sleep problems and learning disabilities and LUTS do not have NE. On the other hand, there are many children with these conditions who also have NE and wet their beds, as was found in our study. It is possible that these children are predisposed to NE due to factors like genetic, family and home characteristics similar to those predictive of MSNE without LUTS or any co-morbid conditions. These children have additional morbidity due to NE which may go undiagnosed and untreated causing unaddressed distress as a result.

Family and home characteristics found to have a significant association included a family history of bedwetting, consanguineous marriage and bullying of the child [4, 5]. Family history of bedwetting in parents is predictive of bedwetting as was found in this study also [4]. Table 3 The frequencies of primary and secondary enuresis, associated variables and risk factors match those of previous studies although data collection was done in primary care family medicine and pediatric clinics and therefore the final prevalence figures may not accurately reflect actual population-based measures.

The study had several potential limitations. Firstly, the study was conducted in an urban city of Pakistan so we cannot comment about the frequencies of NE in rural areas. Secondly, this was conducted in outpatient primary care family medicine and pediatric clinics of public and private hospitals and we did not ask the parents for the reason for attendance in the clinic. Physical examination or investigations regarding their urinary problems were not done on the children. They were grouped into NE categories according to ICCS criteria and terminology based on information and descriptions given by the parents, which is in keeping with the ICCS premise of confirming NE categories/types based on descriptions rather than on pathogenesis. It is possible that there may have been other associated conditions not necessarily ICCS defined in these children like Spina Bifida Occulta (SBO) that could have been diagnosed with investigations only [33]. Further studies that include physical examination and investigations will be able to diagnose more conditions associated with NE beyond the description based ICCS definitions. We did not check for encopresis and asymptomatic bacteriuria, although they are also listed as co-morbids in the ICCS criteria, in the sampled children due to the associated difficulties although we collected data on verbal information about all the remaining LUTS and co-morbids as defined by ICCS. Moreover, since this is a cross sectional study, temporal associations cannot be ruled out. Extrapolation of results to children admitted in hospitals must be done with caution as the data collection was done in outpatient and community-based clinics.

## Conclusion

Nocturnal enuresis as a symptom as described by ICCS is common in children of families visiting outpatient and primary care family medicine and pediatric clinics in Karachi, Pakistan. A number of socio demographic variables are frequently found to be associated with it. It can be present as the only symptom or condition or it can be associated with one or more LUTS or with one or even more than one, co-morbid condition. It is most commonly associated with constipation [31, 32]. It is not known how many parents of children with co-morbidities like constipation and LUTS also report NE as a symptom to their doctors at the time of presentation. This symptom of bedwetting may not always be elicited in history taking of the associated conditions and may not be reported by the family or child due to fear of being shamed, guilt or embarrassment. Therefore, just as it is obligatory to inquire about associated symptoms and conditions in children presenting with the complaint of NE, it is necessary to inquire about NE in children presenting with LUTS, and co-morbid conditions like constipation, UTI, developmental delay, sleep problems and learning disabilities. It is essential to differentiate between the various types of NE as defined by ICCS because the etiology and therefore management of these sub-types is different. This will ensure recognition and treatment of unrecognized morbidity and psychological distress not just in affected children but often the parents, which is especially true for parents of older children. Further research based on ICCS criteria and terminology is necessary to generate reliable and comparable data to be able to address this under recognized condition in children comprehensively and effectively.

## Abbreviations

DSMV: (Diagnostic and Statistical Manual of Mental Disorders, Vth Edition)
ICCS: (International Children’s Continence Society)
LUTS: (Lower urinary tract symptoms)
MSNE: (Monosymptomatic nocturnal enuresis)
NE: (nocturnal enuresis)
NE-LUTS: (Nocturnal enuresis-Lower urinary tract symptoms)
Non-NE: (Non- Nocturnal Enuretics)
SBO: (Spina Bifida Occulta)
UTI: (Urinary tract infection)
WHO: (World Health Organization)
